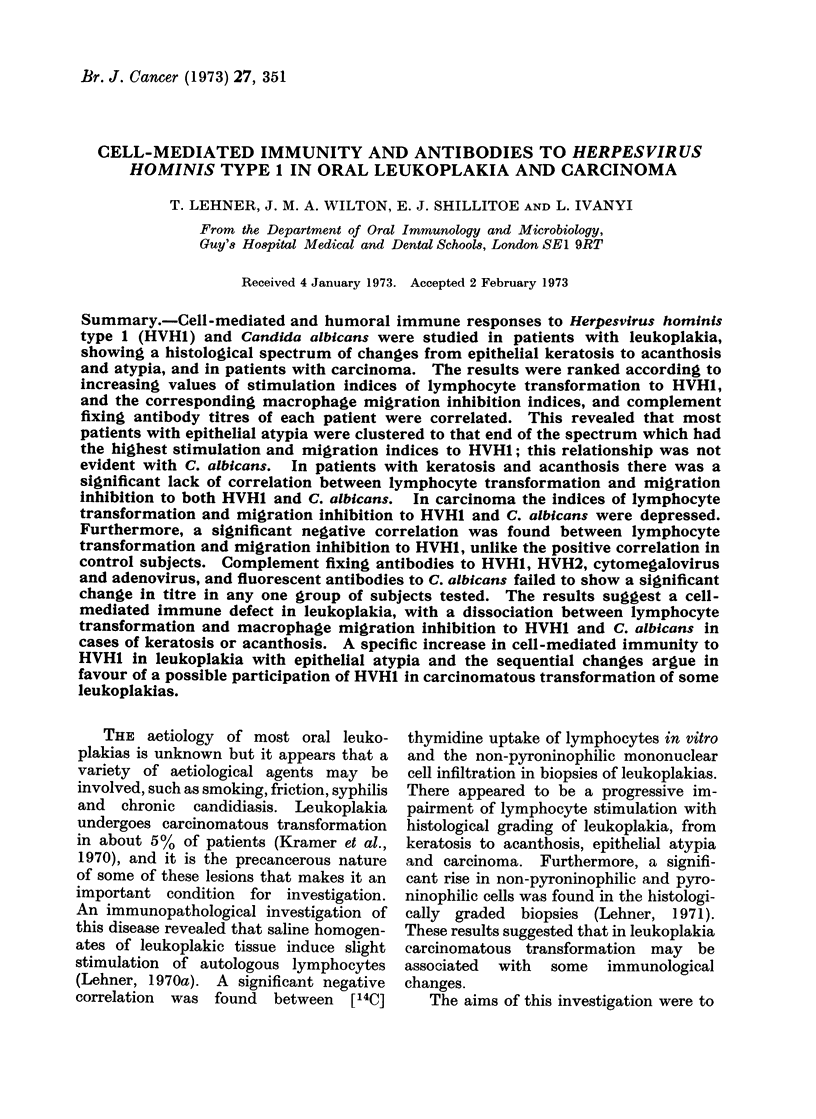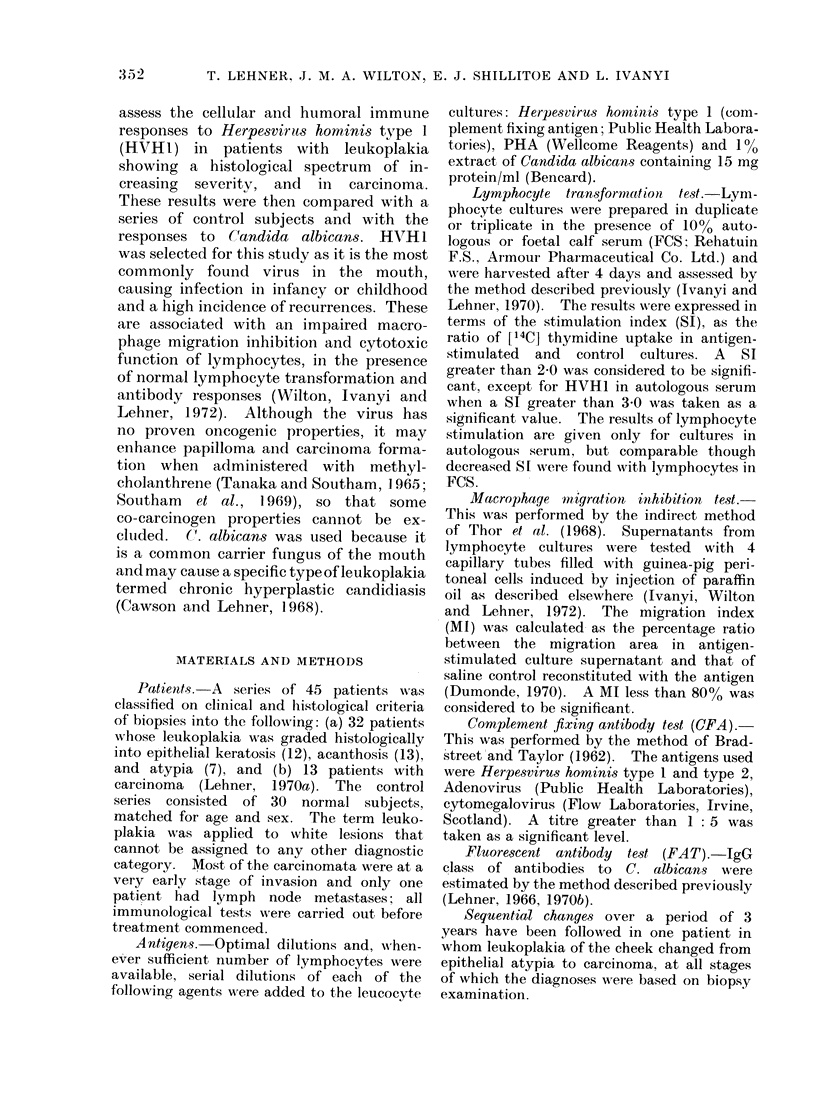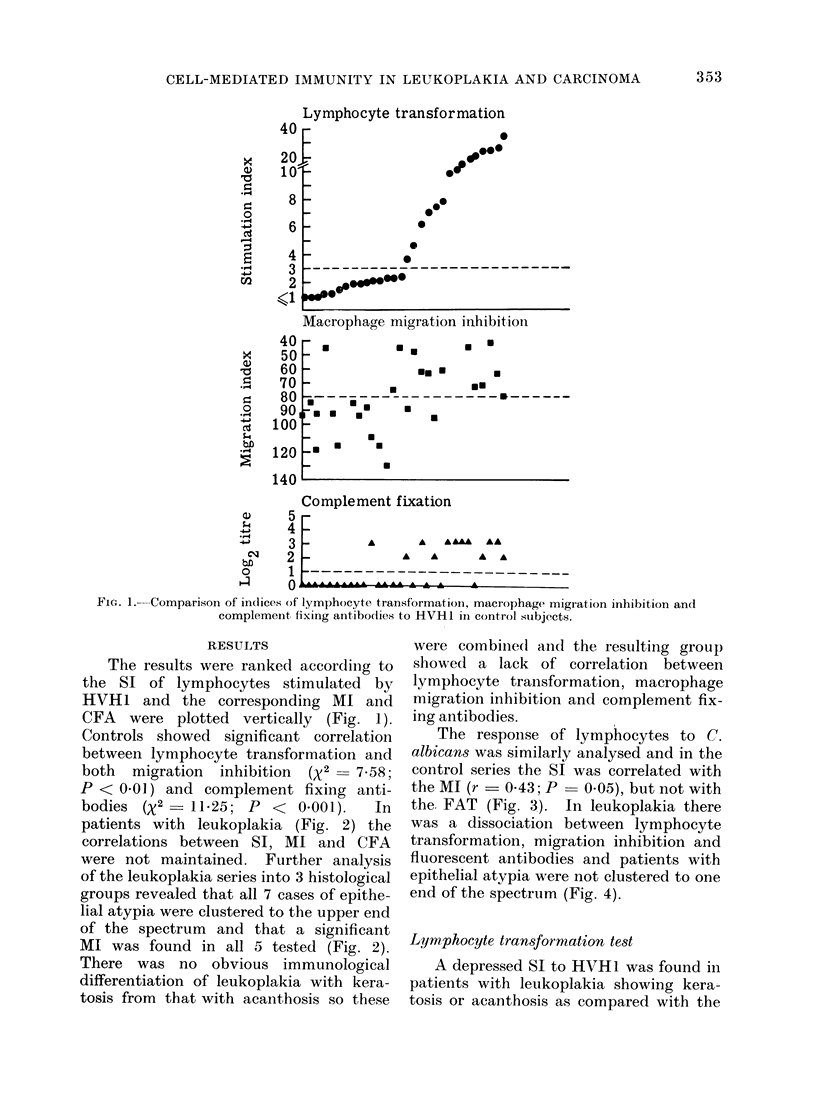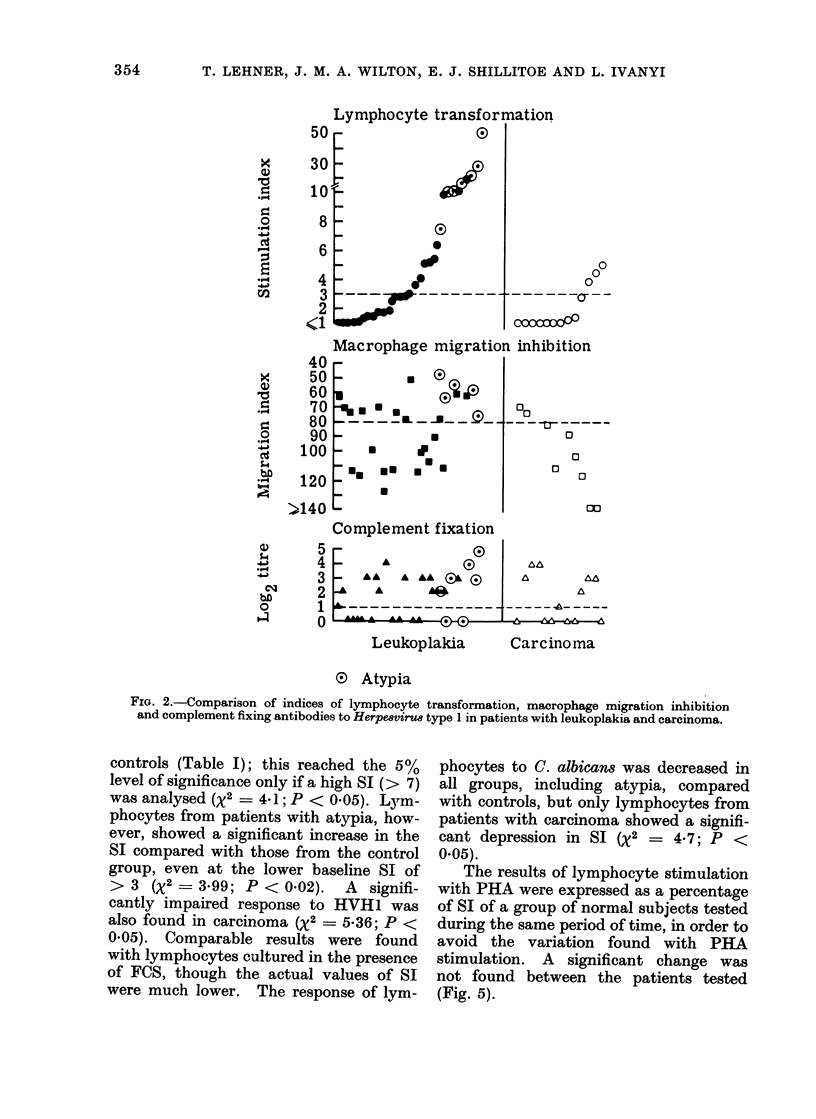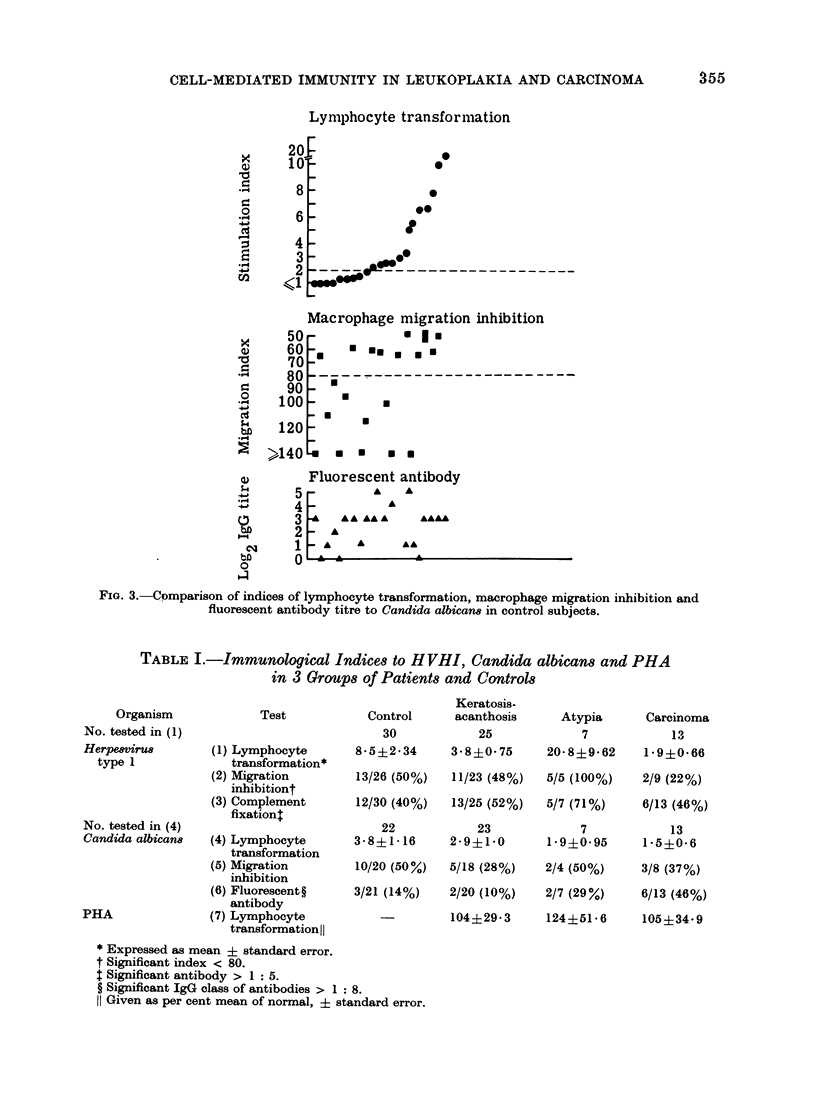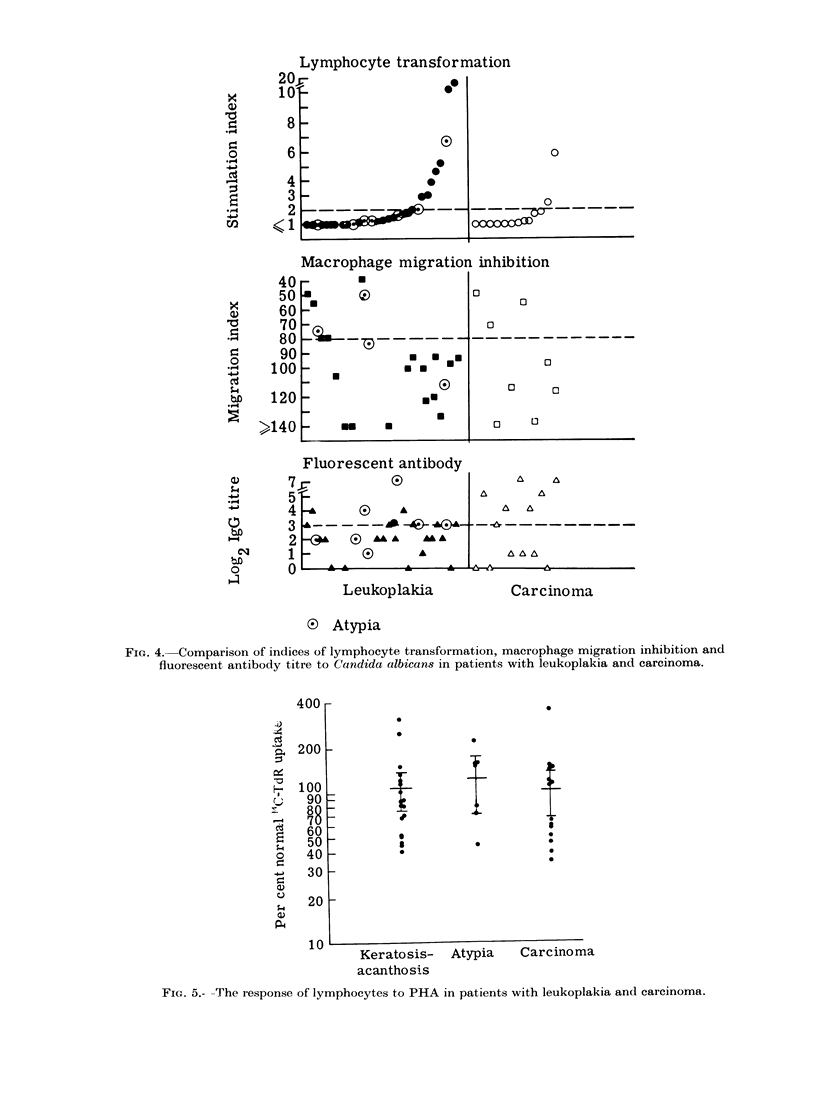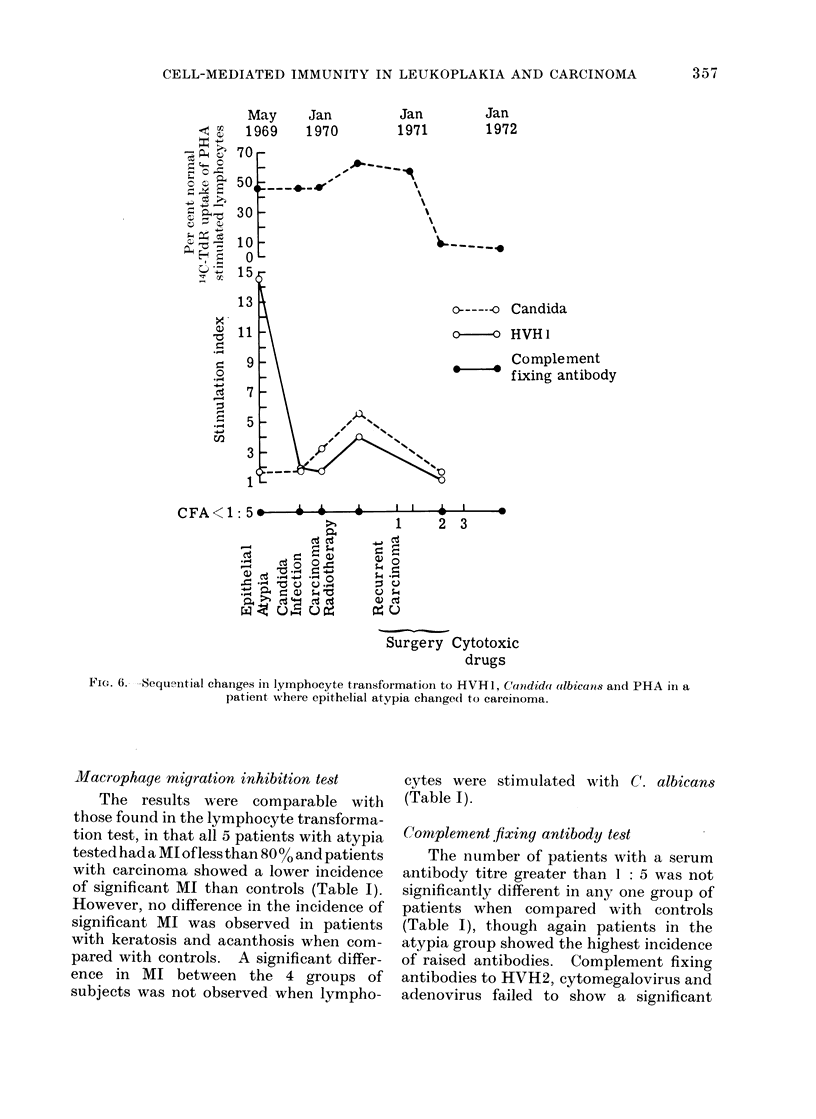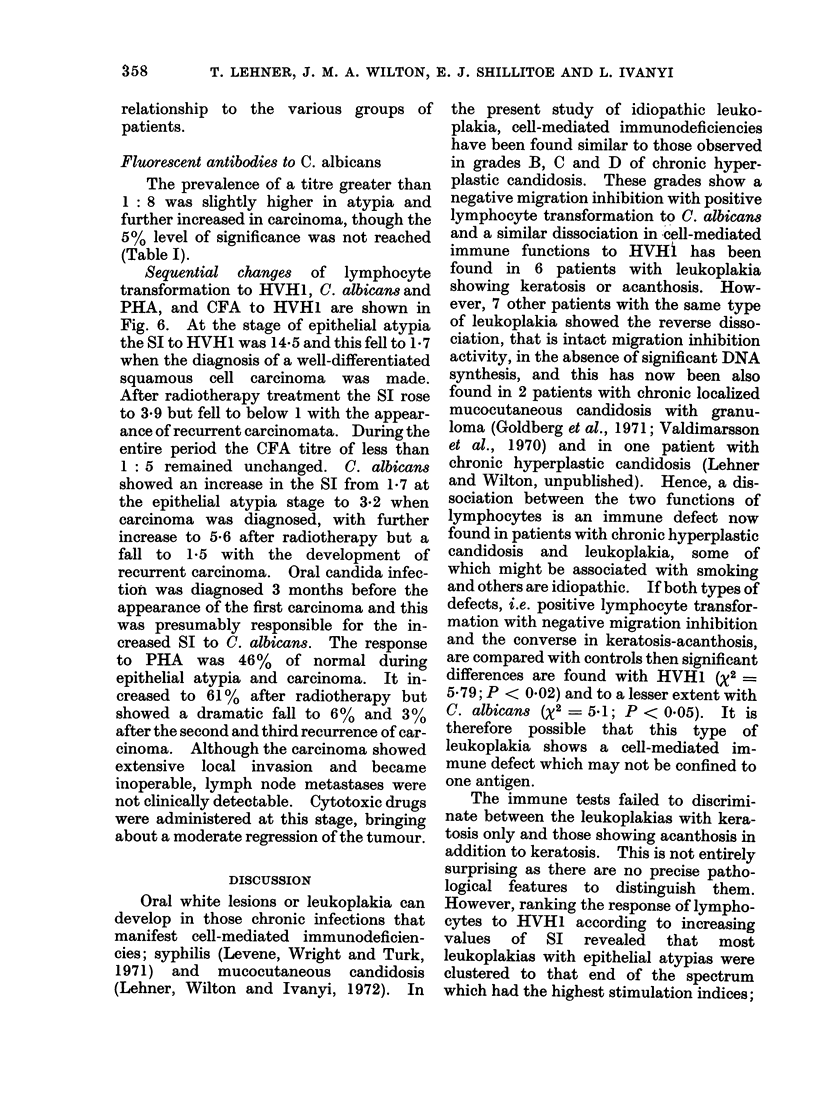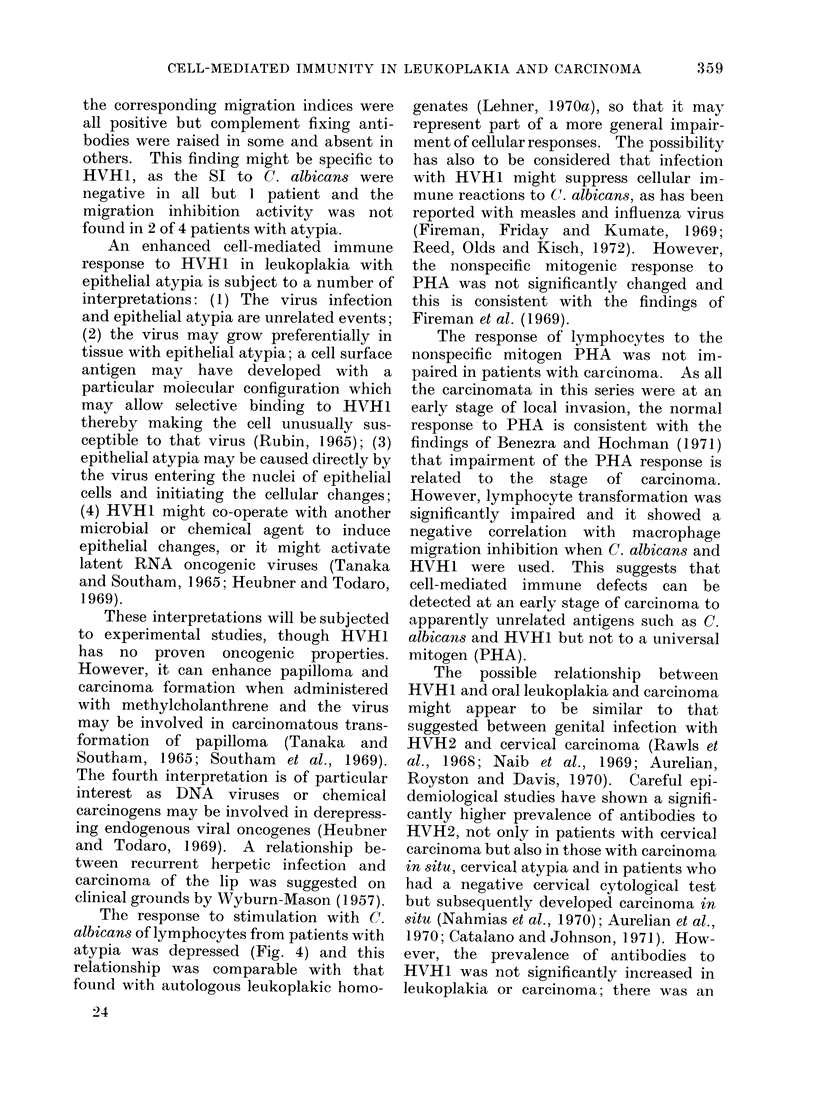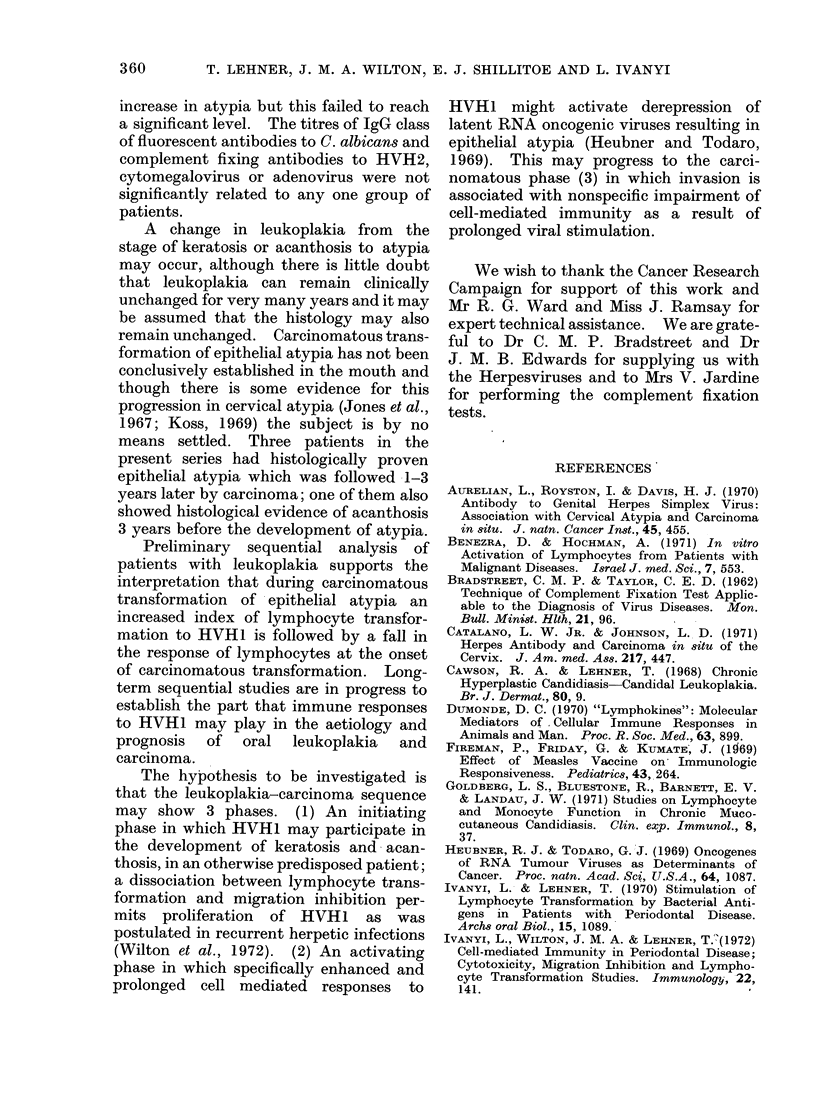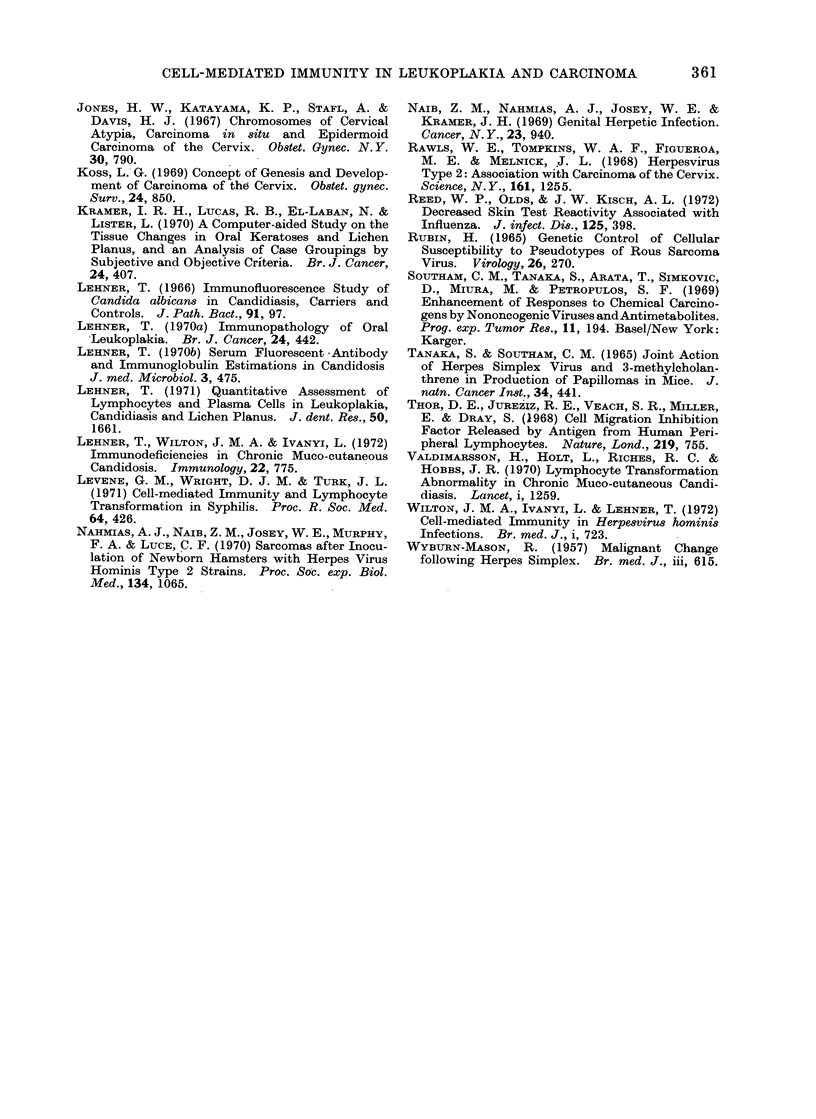# Cell-mediated Immunity and Antibodies to Herpesvirus hominis Type 1 in Oral Leukoplakia and Carcinoma

**DOI:** 10.1038/bjc.1973.43

**Published:** 1973-05

**Authors:** T. Lehner, J. M. A. Wilton, E. J. Shillitoe, L. Ivanyi

## Abstract

Cell-mediated and humoral immune responses to *Herpesvirus hominis* type 1 (HVH1) and *Candida albicans* were studied in patients with leukoplakia, showing a histological spectrum of changes from epithelial keratosis to acanthosis and atypia, and in patients with carcinoma. The results were ranked according to increasing values of stimulation indices of lymphocyte transformation to HVH1, and the corresponding macrophage migration inhibition indices, and complement fixing antibody titres of each patient were correlated. This revealed that most patients with epithelial atypia were clustered to that end of the spectrum which had the highest stimulation and migration indices to HVH1; this relationship was not evident with *C. albicans.* In patients with keratosis and acanthosis there was a significant lack of correlation between lymphocyte transformation and migration inhibition to both HVH1 and *C. albicans.* In carcinoma the indices of lymphocyte transformation and migration inhibition to HVH1 and *C. albicans* were depressed. Furthermore, a significant negative correlation was found between lymphocyte transformation and migration inhibition to HVH1, unlike the positive correlation in control subjects. Complement fixing antibodies to HVH1, HVH2, cytomegalovirus and adenovirus, and fluorescent antibodies to *C. albicans* failed to show a significant change in titre in any one group of subjects tested. The results suggest a cell-mediated immune defect in leukoplakia, with a dissociation between lymphocyte transformation and macrophage migration inhibition to HVH1 and *C. albicans* in cases of keratosis or acanthosis. A specific increase in cell-mediated immunity to HVH1 in leukoplakia with epithelial atypia and the sequential changes argue in favour of a possible participation of HVH1 in carcinomatous transformation of some leukoplakias.


*Br. J. Cancer* (1973) **27**, 351

### CELL-MEDIATED IMMUNITY AND ANTIBODIES TO *HERPESVIRUS HOMINIS* TYPE 1 IN ORAL LEUKOPLAKIA AND CARCINOMA

T. LEHNER, J. M. A. WILTON, E. J. SHILLITOE AND L. IVANYI

*From the Department of Oral Immunology and Microbiology, Guy's Hospital Medical and Dental Schools, London SE1 9RT*

Received 4 January 1973. Accepted 2 February 1973

**Summary.—Cell-mediated and humoral immune responses to *Herpesvirus hominis* type 1 (HVH1) and *Candida albicans* were studied in patients with leukoplakia, showing a histological spectrum of changes from epithelial keratosis to acanthosis and atypia, and in patients with carcinoma. The results were ranked according to increasing values of stimulation indices of lymphocyte transformation to HVH1, and the corresponding macrophage migration inhibition indices, and complement fixing antibody titres of each patient were correlated. This revealed that most patients with epithelial atypia were clustered to that end of the spectrum which had the highest stimulation and migration indices to HVH1; this relationship was not evident with *C. albicans*. In patients with keratosis and acanthosis there was a significant lack of correlation between lymphocyte transformation and migration inhibition to both HVH1 and *C. albicans*. In carcinoma the indices of lymphocyte transformation and migration inhibition to HVH1 and *C. albicans* were depressed. Furthermore, a significant negative correlation was found between lymphocyte transformation and migration inhibition to HVH1, unlike the positive correlation in control subjects. Complement fixing antibodies to HVH1, HVH2, cytomegalovirus and adenovirus, and fluorescent antibodies to *C. albicans* failed to show a significant change in titre in any one group of subjects tested. The results suggest a cell-mediated immune defect in leukoplakia, with a dissociation between lymphocyte transformation and macrophage migration inhibition to HVH1 and *C. albicans* in cases of keratosis or acanthosis. A specific increase in cell-mediated immunity to HVH1 in leukoplakia with epithelial atypia and the sequential changes argue in favour of a possible participation of HVH1 in carcinomatous transformation of some leukoplakias.**

THE aetiology of most oral leukoplakias is unknown but it appears that a variety of aetiological agents may be involved, such as smoking, friction, syphilis and chronic candidiasis. Leukoplakia undergoes carcinomatous transformation in about 5% of patients (Kramer *et al.*, 1970), and it is the precancerous nature of some of these lesions that makes it an important condition for investigation. An immunopathological investigation of this disease revealed that saline homogenates of leukoplakic tissue induce slight stimulation of autologous lymphocytes (Lehner, 1970*a*). A significant negative correlation was found between [$^{14}$C] thymidine uptake of lymphocytes *in vitro* and the non-pyroninophilic mononuclear cell infiltration in biopsies of leukoplakias. There appeared to be a progressive impairment of lymphocyte stimulation with histological grading of leukoplakia, from keratosis to acanthosis, epithelial atypia and carcinoma. Furthermore, a significant rise in non-pyroninophilic and pyroninophilic cells was found in the histologically graded biopsies (Lehner, 1971). These results suggested that in leukoplakia carcinomatous transformation may be associated with some immunological changes.

The aims of this investigation were to assess the cellular and humoral immune responses to *Herpesvirus hominis* type 1 (HVH1) in patients with leukoplakia showing a histological spectrum of increasing severity, and in carcinoma. These results were then compared with a series of control subjects and with the responses to *Candida albicans*. HVH1 was selected for this study as it is the most commonly found virus in the mouth, causing infection in infancy or childhood and a high incidence of recurrences. These are associated with an impaired macrophage migration inhibition and cytotoxic function of lymphocytes, in the presence of normal lymphocyte transformation and antibody responses (Wilton, Ivanyi and Lehner, 1972). Although the virus has no proven oncogenic properties, it may enhance papilloma and carcinoma formation when administered with methylcholanthrene (Tanaka and Southam, 1965; Southam *et al.*, 1969), so that some co-carcinogen properties cannot be excluded. *C. albicans* was used because it is a common carrier fungus of the mouth and may cause a specific type of leukoplakia termed chronic hyperplastic candidiasis (Cawson and Lehner, 1968).

### MATERIALS AND METHODS

*Patients.*—A series of 45 patients was classified on clinical and histological criteria of biopsies into the following: (a) 32 patients whose leukoplakia was graded histologically into epithelial keratosis (12), acanthosis (13), and atypia (7), and (b) 13 patients with carcinoma (Lehner, 1970*a*). The control series consisted of 30 normal subjects, matched for age and sex. The term leukoplakia was applied to white lesions that cannot be assigned to any other diagnostic category. Most of the carcinomata were at a very early stage of invasion and only one patient had lymph node metastases; all immunological tests were carried out before treatment commenced.

*Antigens.*—Optimal dilutions and, whenever sufficient number of lymphocytes were available, serial dilutions of each of the following agents were added to the leucocyte cultures: *Herpesvirus hominis* type 1 (complement fixing antigen; Public Health Laboratories), PHA (Wellcome Reagents) and 1% extract of *Candida albicans* containing 15 mg protein/ml (Bencard).

*Lymphocyte transformation test.*—Lymphocyte cultures were prepared in duplicate or triplicate in the presence of 10% autologous or foetal calf serum (FCS; Rehatuin F.S., Armour Pharmaceutical Co. Ltd.) and were harvested after 4 days and assessed by the method described previously (Ivanyi and Lehner, 1970). The results were expressed in terms of the stimulation index (SI), as the ratio of [$^{14}$C] thymidine uptake in antigen-stimulated and control cultures. A SI greater than 2·0 was considered to be significant, except for HVH1 in autologous serum when a SI greater than 3·0 was taken as a significant value. The results of lymphocyte stimulation are given only for cultures in autologous serum, but comparable though decreased SI were found with lymphocytes in FCS.

*Macrophage migration inhibition test.*—This was performed by the indirect method of Thor *et al.* (1968). Supernatants from lymphocyte cultures were tested with 4 capillary tubes filled with guinea-pig peritoneal cells induced by injection of paraffin oil as described elsewhere (Ivanyi, Wilton and Lehner, 1972). The migration index (MI) was calculated as the percentage ratio between the migration area in antigen-stimulated culture supernatant and that of saline control reconstituted with the antigen (Dumonde, 1970). A MI less than 80% was considered to be significant.

*Complement fixing antibody test (CFA).*—This was performed by the method of Bradstreet and Taylor (1962). The antigens used were *Herpesvirus hominis* type 1 and type 2, Adenovirus (Public Health Laboratories), cytomegalovirus (Flow Laboratories, Irvine, Scotland). A titre greater than 1 : 5 was taken as a significant level.

*Fluorescent antibody test (FAT).*—IgG class of antibodies to *C. albicans* were estimated by the method described previously (Lehner, 1966, 1970*b*).

*Sequential changes* over a period of 3 years have been followed in one patient in whom leukoplakia of the cheek changed from epithelial atypia to carcinoma, at all stages of which the diagnoses were based on biopsy examination.

FIG. 1.—Comparison of indices of lymphocyte transformation, macrophage migration inhibition and complement fixing antibodies to HVH1 in control subjects.

RESULTS

The results were ranked according to the SI of lymphocytes stimulated by HVH1 and the corresponding MI and CFA were plotted vertically (Fig. 1). Controls showed significant correlation between lymphocyte transformation and both migration inhibition ($\chi^2 = 7{\cdot}58$; $P < 0{\cdot}01$) and complement fixing antibodies ($\chi^2 = 11{\cdot}25$; $P < 0{\cdot}001$). In patients with leukoplakia (Fig. 2) the correlations between SI, MI and CFA were not maintained. Further analysis of the leukoplakia series into 3 histological groups revealed that all 7 cases of epithelial atypia were clustered to the upper end of the spectrum and that a significant MI was found in all 5 tested (Fig. 2). There was no obvious immunological differentiation of leukoplakia with keratosis from that with acanthosis so these were combined and the resulting group showed a lack of correlation between lymphocyte transformation, macrophage migration inhibition and complement fixing antibodies.

The response of lymphocytes to *C. albicans* was similarly analysed and in the control series the SI was correlated with the MI ($r = 0{\cdot}43$; $P = 0{\cdot}05$), but not with the FAT (Fig. 3). In leukoplakia there was a dissociation between lymphocyte transformation, migration inhibition and fluorescent antibodies and patients with epithelial atypia were not clustered to one end of the spectrum (Fig. 4).

### *Lymphocyte transformation test*

A depressed SI to HVH1 was found in patients with leukoplakia showing keratosis or acanthosis as compared with the

FIG. 2.—Comparison of indices of lymphocyte transformation, macrophage migration inhibition and complement fixing antibodies to *Herpesvirus* type 1 in patients with leukoplakia and carcinoma.

controls (Table I); this reached the 5% level of significance only if a high SI ($> 7$) was analysed ($\chi^2 = 4{\cdot}1$; $P < 0{\cdot}05$). Lymphocytes from patients with atypia, however, showed a significant increase in the SI compared with those from the control group, even at the lower baseline SI of $> 3$ ($\chi^2 = 3{\cdot}99$; $P < 0{\cdot}02$). A significantly impaired response to HVH1 was also found in carcinoma ($\chi^2 = 5{\cdot}36$; $P < 0{\cdot}05$). Comparable results were found with lymphocytes cultured in the presence of FCS, though the actual values of SI were much lower. The response of lymphocytes to *C. albicans* was decreased in all groups, including atypia, compared with controls, but only lymphocytes from patients with carcinoma showed a significant depression in SI ($\chi^2 = 4{\cdot}7$; $P < 0{\cdot}05$).

The results of lymphocyte stimulation with PHA were expressed as a percentage of SI of a group of normal subjects tested during the same period of time, in order to avoid the variation found with PHA stimulation. A significant change was not found between the patients tested (Fig. 5).

FIG. 3.—Comparison of indices of lymphocyte transformation, macrophage migration inhibition and fluorescent antibody titre to *Candida albicans* in control subjects.

TABLE I.—*Immunological Indices to HVHI, Candida albicans and PHA in 3 Groups of Patients and Controls*

| Organism | Test | Control | Keratosis-acanthosis | Atypia | Carcinoma |
|---|---|---|---|---|---|
| No. tested in (1) | | 30 | 25 | 7 | 13 |
| *Herpesvirus* type 1 | (1) Lymphocyte transformation* | 8·5±2·34 | 3·8±0·75 | 20·8±9·62 | 1·9±0·66 |
| | (2) Migration inhibition† | 13/26 (50%) | 11/23 (48%) | 5/5 (100%) | 2/9 (22%) |
| | (3) Complement fixation‡ | 12/30 (40%) | 13/25 (52%) | 5/7 (71%) | 6/13 (46%) |
| No. tested in (4) | | 22 | 23 | 7 | 13 |
| *Candida albicans* | (4) Lymphocyte transformation | 3·8±1·16 | 2·9±1·0 | 1·9±0·95 | 1·5±0·6 |
| | (5) Migration inhibition | 10/20 (50%) | 5/18 (28%) | 2/4 (50%) | 3/8 (37%) |
| | (6) Fluorescent§ antibody | 3/21 (14%) | 2/20 (10%) | 2/7 (29%) | 6/13 (46%) |
| PHA | (7) Lymphocyte transformation‖ | — | 104±29·3 | 124±51·6 | 105±34·9 |

* Expressed as mean ± standard error.
† Significant index < 80.
‡ Significant antibody > 1 : 5.
§ Significant IgG class of antibodies > 1 : 8.
‖ Given as per cent mean of normal, ± standard error.

FIG. 4.—Comparison of indices of lymphocyte transformation, macrophage migration inhibition and fluorescent antibody titre to *Candida albicans* in patients with leukoplakia and carcinoma.

FIG. 5.—The response of lymphocytes to PHA in patients with leukoplakia and carcinoma.

FIG. 6.—Sequential changes in lymphocyte transformation to HVH1, *Candida albicans* and PHA in a patient where epithelial atypia changed to carcinoma.

*Macrophage migration inhibition test*

The results were comparable with those found in the lymphocyte transformation test, in that all 5 patients with atypia tested had a MI of less than 80% and patients with carcinoma showed a lower incidence of significant MI than controls (Table I). However, no difference in the incidence of significant MI was observed in patients with keratosis and acanthosis when compared with controls. A significant difference in MI between the 4 groups of subjects was not observed when lymphocytes were stimulated with *C. albicans* (Table I).

*Complement fixing antibody test*

The number of patients with a serum antibody titre greater than 1 : 5 was not significantly different in any one group of patients when compared with controls (Table I), though again patients in the atypia group showed the highest incidence of raised antibodies. Complement fixing antibodies to HVH2, cytomegalovirus and adenovirus failed to show a significant relationship to the various groups of patients.

*Fluorescent antibodies to* C. albicans

The prevalence of a titre greater than 1 : 8 was slightly higher in atypia and further increased in carcinoma, though the 5% level of significance was not reached (Table I).

*Sequential changes* of lymphocyte transformation to HVH1, *C. albicans* and PHA, and CFA to HVH1 are shown in Fig. 6. At the stage of epithelial atypia the SI to HVH1 was 14·5 and this fell to 1·7 when the diagnosis of a well-differentiated squamous cell carcinoma was made. After radiotherapy treatment the SI rose to 3·9 but fell to below 1 with the appearance of recurrent carcinomata. During the entire period the CFA titre of less than 1 : 5 remained unchanged. *C. albicans* showed an increase in the SI from 1·7 at the epithelial atypia stage to 3·2 when carcinoma was diagnosed, with further increase to 5·6 after radiotherapy but a fall to 1·5 with the development of recurrent carcinoma. Oral candida infection was diagnosed 3 months before the appearance of the first carcinoma and this was presumably responsible for the increased SI to *C. albicans*. The response to PHA was 46% of normal during epithelial atypia and carcinoma. It increased to 61% after radiotherapy but showed a dramatic fall to 6% and 3% after the second and third recurrence of carcinoma. Although the carcinoma showed extensive local invasion and became inoperable, lymph node metastases were not clinically detectable. Cytotoxic drugs were administered at this stage, bringing about a moderate regression of the tumour.

### DISCUSSION

Oral white lesions or leukoplakia can develop in those chronic infections that manifest cell-mediated immunodeficiencies; syphilis (Levene, Wright and Turk, 1971) and mucocutaneous candidosis (Lehner, Wilton and Ivanyi, 1972). In the present study of idiopathic leukoplakia, cell-mediated immunodeficiencies have been found similar to those observed in grades B, C and D of chronic hyperplastic candidosis. These grades show a negative migration inhibition with positive lymphocyte transformation to *C. albicans* and a similar dissociation in cell-mediated immune functions to HVH1 has been found in 6 patients with leukoplakia showing keratosis or acanthosis. However, 7 other patients with the same type of leukoplakia showed the reverse dissociation, that is intact migration inhibition activity, in the absence of significant DNA synthesis, and this has now been also found in 2 patients with chronic localized mucocutaneous candidosis with granuloma (Goldberg *et al.*, 1971; Valdimarsson *et al.*, 1970) and in one patient with chronic hyperplastic candidosis (Lehner and Wilton, unpublished). Hence, a dissociation between the two functions of lymphocytes is an immune defect now found in patients with chronic hyperplastic candidosis and leukoplakia, some of which might be associated with smoking and others are idiopathic. If both types of defects, *i.e.* positive lymphocyte transformation with negative migration inhibition and the converse in keratosis-acanthosis, are compared with controls then significant differences are found with HVH1 ($\chi^2 = 5{\cdot}79$; $P < 0{\cdot}02$) and to a lesser extent with *C. albicans* ($\chi^2 = 5{\cdot}1$; $P < 0{\cdot}05$). It is therefore possible that this type of leukoplakia shows a cell-mediated immune defect which may not be confined to one antigen.

The immune tests failed to discriminate between the leukoplakias with keratosis only and those showing acanthosis in addition to keratosis. This is not entirely surprising as there are no precise pathological features to distinguish them. However, ranking the response of lymphocytes to HVH1 according to increasing values of SI revealed that most leukoplakias with epithelial atypias were clustered to that end of the spectrum which had the highest stimulation indices; the corresponding migration indices were all positive but complement fixing antibodies were raised in some and absent in others. This finding might be specific to HVH1, as the SI to *C. albicans* were negative in all but 1 patient and the migration inhibition activity was not found in 2 of 4 patients with atypia.

An enhanced cell-mediated immune response to HVH1 in leukoplakia with epithelial atypia is subject to a number of interpretations: (1) The virus infection and epithelial atypia are unrelated events; (2) the virus may grow preferentially in tissue with epithelial atypia; a cell surface antigen may have developed with a particular molecular configuration which may allow selective binding to HVH1 thereby making the cell unusually susceptible to that virus (Rubin, 1965); (3) epithelial atypia may be caused directly by the virus entering the nuclei of epithelial cells and initiating the cellular changes; (4) HVH1 might co-operate with another microbial or chemical agent to induce epithelial changes, or it might activate latent RNA oncogenic viruses (Tanaka and Southam, 1965; Heubner and Todaro, 1969).

These interpretations will be subjected to experimental studies, though HVH1 has no proven oncogenic properties. However, it can enhance papilloma and carcinoma formation when administered with methylcholanthrene and the virus may be involved in carcinomatous transformation of papilloma (Tanaka and Southam, 1965; Southam *et al.*, 1969). The fourth interpretation is of particular interest as DNA viruses or chemical carcinogens may be involved in derepressing endogenous viral oncogenes (Heubner and Todaro, 1969). A relationship between recurrent herpetic infection and carcinoma of the lip was suggested on clinical grounds by Wyburn-Mason (1957).

The response to stimulation with *C. albicans* of lymphocytes from patients with atypia was depressed (Fig. 4) and this relationship was comparable with that found with autologous leukoplakic homogenates (Lehner, 1970*a*), so that it may represent part of a more general impairment of cellular responses. The possibility has also to be considered that infection with HVH1 might suppress cellular immune reactions to *C. albicans*, as has been reported with measles and influenza virus (Fireman, Friday and Kumate, 1969; Reed, Olds and Kisch, 1972). However, the nonspecific mitogenic response to PHA was not significantly changed and this is consistent with the findings of Fireman *et al.* (1969).

The response of lymphocytes to the nonspecific mitogen PHA was not impaired in patients with carcinoma. As all the carcinomata in this series were at an early stage of local invasion, the normal response to PHA is consistent with the findings of Benezra and Hochman (1971) that impairment of the PHA response is related to the stage of carcinoma. However, lymphocyte transformation was significantly impaired and it showed a negative correlation with macrophage migration inhibition when *C. albicans* and HVH1 were used. This suggests that cell-mediated immune defects can be detected at an early stage of carcinoma to apparently unrelated antigens such as *C. albicans* and HVH1 but not to a universal mitogen (PHA).

The possible relationship between HVH1 and oral leukoplakia and carcinoma might appear to be similar to that suggested between genital infection with HVH2 and cervical carcinoma (Rawls *et al.*, 1968; Naib *et al.*, 1969; Aurelian, Royston and Davis, 1970). Careful epidemiological studies have shown a significantly higher prevalence of antibodies to HVH2, not only in patients with cervical carcinoma but also in those with carcinoma *in situ*, cervical atypia and in patients who had a negative cervical cytological test but subsequently developed carcinoma *in situ* (Nahmias *et al.*, 1970); Aurelian *et al.*, 1970; Catalano and Johnson, 1971). However, the prevalence of antibodies to HVH1 was not significantly increased in leukoplakia or carcinoma; there was an increase in atypia but this failed to reach a significant level. The titres of IgG class of fluorescent antibodies to *C. albicans* and complement fixing antibodies to HVH2, cytomegalovirus or adenovirus were not significantly related to any one group of patients.

A change in leukoplakia from the stage of keratosis or acanthosis to atypia may occur, although there is little doubt that leukoplakia can remain clinically unchanged for very many years and it may be assumed that the histology may also remain unchanged. Carcinomatous transformation of epithelial atypia has not been conclusively established in the mouth and though there is some evidence for this progression in cervical atypia (Jones *et al.*, 1967; Koss, 1969) the subject is by no means settled. Three patients in the present series had histologically proven epithelial atypia which was followed 1–3 years later by carcinoma; one of them also showed histological evidence of acanthosis 3 years before the development of atypia.

Preliminary sequential analysis of patients with leukoplakia supports the interpretation that during carcinomatous transformation of epithelial atypia an increased index of lymphocyte transformation to HVH1 is followed by a fall in the response of lymphocytes at the onset of carcinomatous transformation. Long-term sequential studies are in progress to establish the part that immune responses to HVH1 may play in the aetiology and prognosis of oral leukoplakia and carcinoma.

The hypothesis to be investigated is that the leukoplakia–carcinoma sequence may show 3 phases. (1) An initiating phase in which HVH1 may participate in the development of keratosis and acanthosis, in an otherwise predisposed patient; a dissociation between lymphocyte transformation and migration inhibition permits proliferation of HVH1 as was postulated in recurrent herpetic infections (Wilton *et al.*, 1972). (2) An activating phase in which specifically enhanced and prolonged cell mediated responses to HVH1 might activate derepression of latent RNA oncogenic viruses resulting in epithelial atypia (Heubner and Todaro, 1969). This may progress to the carcinomatous phase (3) in which invasion is associated with nonspecific impairment of cell-mediated immunity as a result of prolonged viral stimulation.

We wish to thank the Cancer Research Campaign for support of this work and Mr R. G. Ward and Miss J. Ramsay for expert technical assistance. We are grateful to Dr C. M. P. Bradstreet and Dr J. M. B. Edwards for supplying us with the Herpesviruses and to Mrs V. Jardine for performing the complement fixation tests.

### REFERENCES

Aurelian, L., Royston, I. & Davis, H. J. (1970) Antibody to Genital Herpes Simplex Virus: Association with Cervical Atypia and Carcinoma *in situ*. *J. natn. Cancer Inst.*, **45**, 455.

Benezra, D. & Hochman, A. (1971) *In vitro* Activation of Lymphocytes from Patients with Malignant Diseases. *Israel J. med. Sci.*, **7**, 553.

Bradstreet, C. M. P. & Taylor, C. E. D. (1962) Technique of Complement Fixation Test Applicable to the Diagnosis of Virus Diseases. *Mon. Bull. Minist. Hlth*, **21**, 96.

Catalano, L. W. Jr. & Johnson, L. D. (1971) Herpes Antibody and Carcinoma *in situ* of the Cervix. *J. Am. med. Ass.* **217**, 447.

Cawson, R. A. & Lehner, T. (1968) Chronic Hyperplastic Candidiasis—Candidal Leukoplakia. *Br. J. Dermat.*, **80**, 9.

Dumonde, D. C. (1970) "Lymphokines": Molecular Mediators of Cellular Immune Responses in Animals and Man. *Proc. R. Soc. Med.*, **63**, 899.

Fireman, P., Friday, G. & Kumate, J. (1969) Effect of Measles Vaccine on Immunologic Responsiveness. *Pediatrics*, **43**, 264.

Goldberg, L. S., Bluestone, R., Barnett, E. V. & Landau, J. W. (1971) Studies on Lymphocyte and Monocyte Function in Chronic Mucocutaneous Candidiasis. *Clin. exp. Immunol.*, **8**, 37.

Heubner, R. J. & Todaro, G. J. (1969) Oncogenes of RNA Tumour Viruses as Determinants of Cancer. *Proc. natn. Acad. Sci, U.S.A.*, **64**, 1087.

Ivanyi, L. & Lehner, T. (1970) Stimulation of Lymphocyte Transformation by Bacterial Antigens in Patients with Periodontal Disease. *Archs oral Biol.*, **15**, 1089.

Ivanyi, L., Wilton, J. M. A. & Lehner, T. (1972) Cell-mediated Immunity in Periodontal Disease; Cytotoxicity, Migration Inhibition and Lymphocyte Transformation Studies. *Immunology*, **22**, 141.

JONES, H. W., KATAYAMA, K. P., STAFL, A. & DAVIS, H. J. (1967) Chromosomes of Cervical Atypia, Carcinoma *in situ* and Epidermoid Carcinoma of the Cervix. *Obstet. Gynec. N.Y.* **30**, 790.

KOSS, L. G. (1969) Concept of Genesis and Development of Carcinoma of the Cervix. *Obstet. gynec. Surv.*, **24**, 850.

KRAMER, I. R. H., LUCAS, R. B., EL-LABAN, N. & LISTER, L. (1970) A Computer-aided Study on the Tissue Changes in Oral Keratoses and Lichen Planus, and an Analysis of Case Groupings by Subjective and Objective Criteria. *Br. J. Cancer*, **24**, 407.

LEHNER, T. (1966) Immunofluorescence Study of *Candida albicans* in Candidiasis, Carriers and Controls. *J. Path. Bact.*, **91**, 97.

LEHNER, T. (1970*a*) Immunopathology of Oral Leukoplakia. *Br. J. Cancer*, **24**, 442.

LEHNER, T. (1970*b*) Serum Fluorescent Antibody and Immunoglobulin Estimations in Candidosis *J. med. Microbiol.* **3**, 475.

LEHNER, T. (1971) Quantitative Assessment of Lymphocytes and Plasma Cells in Leukoplakia, Candidiasis and Lichen Planus. *J. dent. Res.*, **50**, 1661.

LEHNER, T., WILTON, J. M. A. & IVANYI, L. (1972) Immunodeficiencies in Chronic Muco-cutaneous Candidosis. *Immunology*, **22**, 775.

LEVENE, G. M., WRIGHT, D. J. M. & TURK, J. L. (1971) Cell-mediated Immunity and Lymphocyte Transformation in Syphilis. *Proc. R. Soc. Med.* **64**, 426.

NAHMIAS, A. J., NAIB, Z. M., JOSEY, W. E., MURPHY, F. A. & LUCE, C. F. (1970) Sarcomas after Inoculation of Newborn Hamsters with Herpes Virus Hominis Type 2 Strains. *Proc. Soc. exp. Biol. Med.*, **134**, 1065.

NAIB, Z. M., NAHMIAS, A. J., JOSEY, W. E. & KRAMER, J. H. (1969) Genital Herpetic Infection. *Cancer, N.Y.*, **23**, 940.

RAWLS, W. E., TOMPKINS, W. A. F., FIGUEROA, M. E. & MELNICK, J. L. (1968) Herpesvirus Type 2: Association with Carcinoma of the Cervix. *Science, N.Y.*, **161**, 1255.

REED, W. P., OLDS, & J. W. KISCH, A. L. (1972) Decreased Skin Test Reactivity Associated with Influenza. *J. infect. Dis.*, **125**, 398.

RUBIN, H. (1965) Genetic Control of Cellular Susceptibility to Pseudotypes of Rous Sarcoma Virus. *Virology*, **26**, 270.

SOUTHAM, C. M., TANAKA, S., ARATA, T., SIMKOVIC, D., MIURA, M. & PETROPULOS, S. F. (1969) Enhancement of Responses to Chemical Carcinogens by Nononcogenic Viruses and Antimetabolites. *Prog. exp. Tumor Res.*, **11**, 194. Basel/New York: Karger.

TANAKA, S. & SOUTHAM, C. M. (1965) Joint Action of Herpes Simplex Virus and 3-methylcholanthrene in Production of Papillomas in Mice. *J. natn. Cancer Inst.*, **34**, 441.

THOR, D. E., JUREZIZ, R. E., VEACH, S. R., MILLER, E. & DRAY, S. (1968) Cell Migration Inhibition Factor Released by Antigen from Human Peripheral Lymphocytes. *Nature, Lond.*, **219**, 755.

VALDIMARSSON, H., HOLT, L., RICHES, R. C. & HOBBS, J. R. (1970) Lymphocyte Transformation Abnormality in Chronic Muco-cutaneous Candidiasis. *Lancet*, i, 1259.

WILTON, J. M. A., IVANYI, L. & LEHNER, T. (1972) Cell-mediated Immunity in *Herpesvirus hominis* Infections. *Br. med. J.*, i, 723.

WYBURN-MASON, R. (1957) Malignant Change following Herpes Simplex. *Br. med. J.*, iii, 615.